# Exosomes: A Promising Strategy for Repair, Regeneration and Treatment of Skin Disorders

**DOI:** 10.3390/cells12121625

**Published:** 2023-06-14

**Authors:** Mario Adrián Tienda-Vázquez, Juan Manuel Hanel, Elsa Margarita Márquez-Arteaga, Ana Paola Salgado-Álvarez, Christian Quintus Scheckhuber, José Rafael Alanis-Gómez, Janette Ivone Espinoza-Silva, Manuel Ramos-Kuri, Fabiola Hernández-Rosas, Elda M. Melchor-Martínez, Roberto Parra-Saldívar

**Affiliations:** 1 School of Engineering and Sciences, Tecnologico de Monterrey, Monterrey 64849, Mexico; a01204468@tec.mx (M.A.T.-V.); c.scheckhuber@tec.mx (C.Q.S.); 2 Biomedical Engineering Program, Faculty of Engineering, Anahuac Queretaro University, Querétaro 76246, Mexico; juan.hanel15@anahuac.mx (J.M.H.); elsa.marquez01@anahuac.mx (E.M.M.-A.); ana.salgado96@anahuac.mx (A.P.S.-Á.); rafael.alanis@anahuac.mx (J.R.A.-G.); fabiola.hernandez86@anahuac.mx (F.H.-R.); 3 School of Engineering and Sciences, Campus Mexico City, Tecnologico de Monterrey, Mexico City 14380, Mexico; 4 Division Research and Postgraduate Division, Faculty of Engineering, Autonomous University of Querétaro, Querétaro 76010, Mexico; 5 Department of Internal Medicine, General Hospital of Querétaro, Querétaro 76175, Mexico; jannymed07@gmail.com; 6 Escuela de Medicina y Ciencias de la Salud, Tecnologico de Monterrey, Monterrey 64710, Mexico; manuel.ramos@tec.mx; 7 Research Center, Anahuac Queretaro University, Querétaro 76246, Mexico; 8 Institute of Advanced Materials for Sustainable Manufacturing, Tecnologico de Monterrey, Monterrey 64849, Mexico

**Keywords:** exosomes, mesenchymal stem cell-derived, wound care, skin damage, diabetic wounds

## Abstract

The skin is the organ that serves as the outermost layer of protection against injury, pathogens, and homeostasis with external factors; in turn, it can be damaged by factors such as burns, trauma, exposure to ultraviolet light (UV), infrared radiation (IR), activating signaling pathways such as Toll-like receptors (TLR) and Nuclear factor erythroid 2-related factor 2 (NRF2), among others, causing a need to subsequently repair and regenerate the skin. However, pathologies such as diabetes lengthen the inflammatory stage, complicating the healing process and, in some cases, completely inhibiting it, generating susceptibility to infections. Exosomes are nano-sized extracellular vesicles that can be isolated and purified from different sources such as blood, urine, breast milk, saliva, urine, umbilical cord bile cells, and mesenchymal stem cells. They have bioactive compounds that, thanks to their paracrine activity, have proven to be effective as anti-inflammatory agents, inducers of macrophage polarization and accelerators of skin repair and regeneration, reducing the possible complications relating to poor wound repair, and prolonged inflammation. This review provides information on the use of exosomes as a promising therapy against damage from UV light, infrared radiation, burns, and skin disorders.

## 1. Introduction

**Figure 1:**
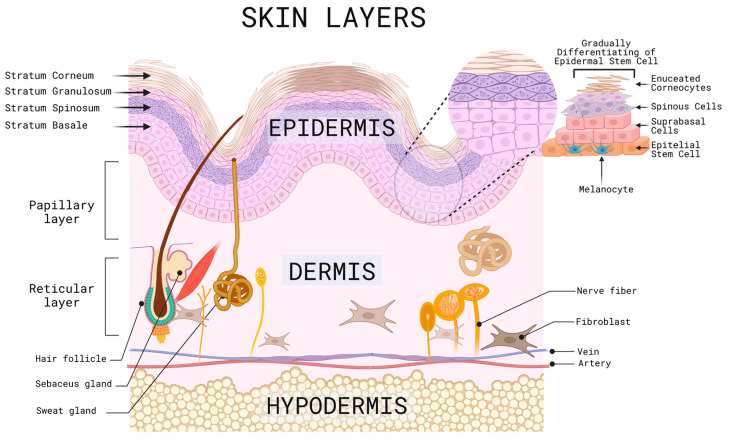
Representative scheme of the different layers, sublayers and cells of the skin. Created with BioRender.com (accessed on 2 June 2023).

The skin is known as the largest organ, representing about eight percent of the body’s weight [1]. The skin covers the surface of the body, belonging to the integumentary system, and is derived from the ectoderm. Its main function is to act as a physical barrier against the external environment [2], protecting internal organs, bones, muscles, and other soft structures from pathogenic microorganisms, chemicals, loss of water, electrolytes, and thermal homeostasis has an average surface area of 1.5 m^2^ to 2.0 m^2^ and an average thickness of 0.3 cm [2,3,4]. This organ is divided into different layers, and in order from the outermost to the innermost are the epidermis, dermal-epidermal junction, dermis, and subcutaneous cell tissue, mostly known as hypodermis, which, in turn, are subdivided into the sublayers described below (Figure 1) [3,4].

The epidermis is the outermost layer of the skin, which makes it susceptible to the environment, with a risk of injury from factors such as UV solar radiation, trauma, ulcers, and surgical procedures, among others. It is composed of stratified squamous epithelium, melanocytes, and keratinocytes, which are in constant change and renewal and are induced to multiply by tissue injury or after a specific stimulation that requires tissue restoration, gradually differentiating from stem cells into suprabasal cells, spinous cells, cells with keratohyalin granules and finally enucleated corneocytes, as they migrate to the skin’s surface and accumulate keratin that is released daily. This process gives rise to several sublayers, including the stratum corneum, which is the outermost layer where desquamation occurs due to the detachment of these cells, followed by the stratum spinosum and the stratum granulosum, and finally, the basal layer or stratum germinativum, which is the deepest layer where the melanocyte precursor melanoblasts are located; these are in contact with keratinocytes through their dendrites, which are in charge of producing melanin, that determine the color of the skin, hair, and provides protection against UV rays [5,6,7,8].

The dermal-epidermal junction is a membrane that lies between the basal membrane of the epidermis and the papillary layer of the dermis and is composed of keratinocytes and fibroblasts, sheets and filaments of keratin, collagen IV, and proteoglycans. Its functions are to provide support, scaffolding, and adhesion to its underlying layers, in addition to participating in tissue repair processes in healing [9].

The dermis is the middle layer of the skin; it has a thickness of 2 to 4 mm, composed of connective tissue of collagen, elastin and amorphous filamentous reticular fibers, blood vessels, lymphatics, hair follicles, cutaneous nerve receptors, endothelial cells, and cells of Schwann [10,11,12]. It has two sublayers; the superficial dermis, also called the papillary layer, contains an abundant extracellular matrix made up of specialized proteins, glycosaminoglycans, and carbohydrates, while the second sublayer, the reticular layer, made up of the middle dermis and the deep or basal dermis, are cells such as fibroblasts that give firmness and hydration to the skin. In case of an injury at this level, there is no regeneration; instead, there is a repair process which will be described later [13,14]. Finally, the hypodermis or subcutaneous cell tissue is composed mainly of fat tissue and blood vessels [15].

**Figure 2:**
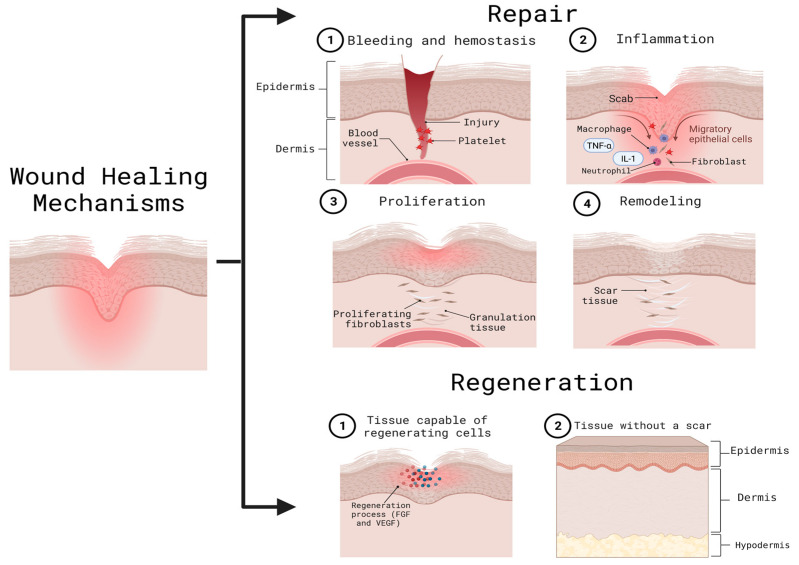
Wound healing mechanisms. The repair mechanism is carried out through hemostasis (an inflammatory phase) re-epithelialization or proliferation, and tissue remodeling. After this, the regeneration process is responsible for the formation of new cells without leaving an appreciable scar. Both repair and regeneration include the action of cytokines, chemokines, and growth factors. FGF: fibroblast growth factor; IL-1: interleukin-1; TNFα: tumor necrosis factor; VEGF: vascular endothelial growth. Created with BioRender.com (accessed on 2 June 2023).

When an injury occurs, time plays a fundamental role in the defense against pathogens, repair, and epithelial regeneration [16]. To repair the lesions, it is necessary to involve other cellular mechanisms, such as chemokines, cytokines, tumor necrosis factor (TNFα), and interleukin-1 (IL-1) as an initial step, and subsequently, growth factors, such as the fibroblast growth factor (FGF) and vascular endothelial growth (VEGF) [1]. Adult skin has two mechanisms to deal with wounds: regeneration and repair. When regeneration occurs, the tissue can replace damaged cells with new ones without leaving a noticeable scar; on the other hand, in the repair process, the damaged tissue presents an inability to produce normal cells resulting in the formation of scars (Figure 2) [15]. Moreover, an ideally healed wound is known to present a normal anatomical structure, function, and appearance after some time, while a minimally healed wound presents a normal anatomical structure but without a functional result [2].

The healing process starts right after an injury is made. It begins with clot formation by the platelets, better known as hemostasis, followed by an inflammatory phase involving an immune response, where neutrophils remove necrotic tissue and bacteria, and macrophages facilitate cytokine and growth factor transformation [2]. Over the following days, a proliferation phase called the re-epithelization process occurs; this consists of the proliferation, migration, differentiation, and stratification of keratinocytes to the center of the trauma creating a new epithelium. The last stage of this process, which can vary in duration between weeks and years, is called the remodeling phase, and this is characterized by the presence of fibroblasts and collagen, fibers which lead to the maturation of the wound and achieve the tissue’s complete recovery [1,2]. As previously described, the re-epithelialization process is essential when a wound occurs as cellular exhaustion is generated; this causes a stimulus to recruit hair follicle cells and produce epidermal cells [5].

Extracellular vesicles are responsible for various physiological processes such as intercellular communication and tissue repair. There is a subset of extracellular vesicles called exosomes, whose size ranges from 30 to 200 nm, which can carry different biomolecules, such as proteins, lipids, and nucleic acids (RNA and DNA). These biomolecules are specific cargoes that depend on the parental cell, that is, the cell that produced the exosomes. Exosomes act as messengers, transporting the biomolecules that can selectively be taken up by recipient cells in order to influence various cellular processes, such as gene expression, cell-to-cell signaling, immune response, and tissue repair. They have the advantage of being able to travel through body fluids such as blood, saliva, and urine, among others, allowing them to reach distant cells and tissues [17,18,19,20,21,22]. Due to their ability to transfer biological information and mediate tissue repair processes, exosomes are a promising strategy to repair, regenerate, and treat skin disorders.

In this review, we present a comprehensive and novel understanding of the emerging roles and therapeutic targets of exosomes in skin homeostasis from the perspective of the signaling pathways involved in cell repair and regeneration processes of general skin wounds, diabetic skin wounds, and UV-induced skin wounds.

## 2. Mechanisms of Skin Related Injury and Repair

### 2.1. Skin Response and Inflammation to Ultraviolet (UV) Light

UVB radiation (280–315 nm) causes the formation of cyclobutene and pyrimidine dimers, using the pyrimidine bases to form covalent links among other toxic and mutagenic photoproducts that block transcription and replication, leading to mutations. To repair the damage caused by UV light in DNA, there is the mechanism of nucleotide excision repair (NER). As an evolutionarily conserved pathway, DNA repairs depend on the degree of damage in the double helix, which can potentially inhibit the processes of replication and transcription [23]. Extensive DNA damage and mutation have also been proven to be related to susceptibility to cancer in UVB-irradiated mice [23].

Oxidative stress damage accelerates skin aging, manifesting as wrinkles, dryness, and, in severe cases, skin cancer. Excessive exposure to UV radiation generates the production of metalloproteinases (MMP), which are responsible for the degradation of the dermal extracellular matrix; secondary to this, the synthesis of collagen and elastin are comprised, as well as the integrity and function of the dermis and epidermis layers, which are lost [24]. The excessive production of reactive oxygen species (ROS) caused by exposure to radiation also leads to an increase in inflammatory factors such as p-NF-κβ and Iκβα, causing severe skin inflammation [25].

UV exposure positively regulates mitogen-activated protein kinase (MAPK), and other triggering factors can include human beta-defensin 2 (hBD2), MAPK, which, when activated, inhibits the cytokine transforming growth factor (TGF-b), degrading collagen [26] hBD2, which generates ROS and in turn is regulated by activating protein 1 (AP-1), inhibiting the synthesis of collagen. AP-1 and singlet molecular oxygen (^1^O_2_) regulates the expression of the c-Jun-N-terminal kinase (JNK) and p38 proteins, which increase the levels of cyclooxygenase-2 (COX-2). It participates in the production of prostaglandins and tumors and increases interleukin-8 (IL-8), IL-1, IL-6, IL-10, IL-12, and TNF-alpha and VEGF; the latter is involved in angiogenesis in keratinocytes [26].

### 2.2. Skin Response to Infrared Radiation (IR)

Not only UV can elicit a multitude of responses from exposed skin. IR, which is converted to heat in the skin, has been known to cause a variety of cellular reactions, some of which are briefly described here. Sunlight consists of different qualities of IR, namely IR-A (760–1440 nm), IR-B (1440–3000 nm), and IR-C (3000 nm–1 mm) [27]. The abilities to penetrate the skin are different for the aforementioned types of IR [28,29]: IR-A can reach a deeper subcutis layer, passing through both the epidermis and the dermis, whereas IR-B is absorbed in the dermis and IR-C in the epidermal layer of the skin. Direct IR irradiation can elevate the skin temperature up to 40 °C, which leads to numerous cellular responses [30]. However, it should be kept in mind that the individual contributions of IR and heat can be clearly distinguished in terms of the cellular responses that are evoked. For example, IR irradiation can alter the expression levels of MMP [31,32] and collagen [32]. Multiple IR irradiations lead to a pronounced increase in the MMP-1 expression in human skin which might contribute to premature aging [32]. Additionally, these treatments can lead to a reduction in procollagen type I expression levels [32], further establishing a mechanistic link between IR and the photoaging of the skin. In addition, IR-A irradiation leads to the induction of dermal angiogenesis by decreasing the ratio of VEGF and the endogenous angiogenic inhibitor TSP-2 [33]. This leads to the reddening of the skin and its faster aging. Further changes, such as mast cell number increase and tryptase expression, have been reported (see [29] for an overview of this topic).

An IR-mediated heat increase in the skin also leads to a plethora of cellular responses, some of which are described here briefly. It was reported that two proteins of skin, tropoelastin and fibrillin-1, were differentially regulated in terms of mRNA and protein levels [34]. Tropoelastin is the soluble precursor of elastin which is the principal component of elastic fibers in the skin, providing both flexibility and resilience [35], whereas fibrillin-1 is an extracellular glycoprotein that is an important structural component of microfibrils [36]. Both mRNA and protein levels of tropoelastin, and fibrillin-1 were found to increase after heat treatment in the epidermis. However, fibrillin-1 expression was reduced in the dermis layer of the skin [34]. The authors of this study concluded that “the abnormal production of tropoelastin and fibrillin by heat, such as UV, in human skin and their degradation by various MMPs, such as MMP-12, may add to the accumulation of elastotic material in photoaged skin” [34].

Similarly, UV heat can also induce changes in DNA; 90 min at 43 °C led to the robust appearance of the oxidative damage marker 8-oxo-dG in both the epidermis and dermis 24 h post the heat-shock, suggesting that damage to DNA occurred. Cho et al. found no evidence that thymidine dimers were being formed [29], which is a hallmark effect after UV irradiation [37]. This finding indicated that UV and IR/heat-mediated DNA damage needed to be carefully distinguished.

It should be stressed that the IR-C treatment of skin can also have clear beneficial effects if applied under strictly controlled conditions. One example came from research that studied the effects of FIR (far infrared) therapy targeted at inhibiting UV-B-mediated photoaging in both hairless mice and NIH 3T3 mouse embryonic fibroblasts [38]. In this study, FIR was able to counteract the UV-B-mediated induction of MMP-1 and MMP-9, which are contributors to skin photoaging. Other interesting effects are increased signaling via the TGF-β/Smad pathway after FIR treatment which can lead to a boost in procollagen type I levels. Additionally, autophagy as a cellular pathway for maintaining homeostasis was found to be induced in an FIR-dependent manner, blocking the hyperproliferative response of the epidermis to UV-B [38]. These results are an example that IR irradiation can be both beneficial and damaging to the skin and that its wavelength and the context under which it occurs are important to recognize [39].

### 2.3. Skin Response to Diabetic Wounds

Diabetes mellitus is a chronic and systemic condition, the effects of which increment over time, such as retinopathy, neuropathy, and even the rate of skin injuries, which are characterized by a distinct lack of healing, reduced epithelization, and prolonged inflammation [23]. According to Quondamatteo, [40] more than 1/3 of diabetes patients present complications relating to cutaneous wounds, which, in severe cases, such as non-healing ulcers, can result in a serious infection and even the amputation of the affected extremity [24]. Of all the non-healing diabetic foot ulcers, 25% require an amputation [25].

Wound healing is a fundamental physiological process as a reaction to internal and external damage to tissue, including skin [23]. Macrophages are a fundamental component of the wound healing process, as they serve as both pro and anti-inflammatory factors. The process starts with an M1 phenotype macrophage enhancing the inflammation of the wounded area in an effort to clear pathogens and cell debris. Diabetes disrupts this process; hyperglycemia can affect immune system cells, such as macrophages, prolonging the inflammation stage, increasing the risk of infection, and stopping the damaged tissue from entering the remodeling stage of healing [41]. Substances such as metalloproteinases and pro-inflammatory molecules reduce the migration rate and the proliferation of fibroblasts, endothelial cells, and keratinocytes, which are fundamental for wound repair [42]. Additionally, hyperglycemia caused by diabetes has been shown to alter the properties of collagen, decreasing its solubility and flexibility (fibrosis) and resulting in a rigid material. Keratinocytes are also affected as their proliferation and rate of differentiation decrease dramatically. Therefore, factors such as fibrosis, decreased keratinocytes, and maintaining sustained inflammation derived from the M1 macrophage phenotype lengthen the inflammatory stage and hinder tissue regeneration [43].

### 2.4. Skin Response to Burn Wounds & Macrophage-Mediated Skin Wound Healing

Damage to the skin caused by heat, chemicals, UV rays, radiation, friction, electric current, and cold are commonly known as burn wounds. These wounds can cause different systemic responses, such as an increment in the capillaries’ permeability, bronchospasms, problems with contractibility in the heart muscle, and an increment in metabolism, among others. Even though a wound of this type involves tissue destruction, it can be associated with multiple physiological and pathophysiological responses. The immune response is the foundation for its repair, triggering proinflammatory and anti-inflammatory phases to conserve the body’s homeostasis. When this response is compromised, different approaches to healing these wounds must be found. Additionally, diverse treatments are determined by the type of burn injury and the class, which range from superficial or first-degree burns to fourth degrees that involve an injury to deeper tissues. Severe wounds can cause a complex pattern of response that can last from days to years and are mainly uncontrolled, causing a generalized catabolic state and delayed healing or even contributing to organ dysfunction and death [44,45].

Macrophages are multitasked cells that work as the primary source of defense against foreign pathogens that enter the organism as an effect of a cutaneous wound. M1 macrophages are associated with pro-inflammatory functions and are fundamental in the elimination of potentially harmful pathogens. M2 macrophages, on the other hand, have anti-inflammatory functions, which are heavily related to the processes of tissue repair [46].

Different factors, such as the presence of a wound or a pathogen, can trigger the formation of a specific type of macrophage or the polarization of an M1 macrophage into an M2 or vice versa, considering the organism’s requirements. This effect is induced by activators such as IL-4, which can induce macrophage polarization to M2, and endotoxin (lipopolysaccharide or LPS), which induces polarization to the M1 phenotype [46]. These activation factors are used to result in the type of macrophage produced. As such, modifying the environment in which the macrophages work can result in the polarization of the specific phenotypes, leading to the desired pro or anti-inflammatory responses.

When tissue is infected, macrophages polarize to the M1 phenotype with the main goal of eliminating foreign pathogens and producing a pro-inflammatory response. They are then polarized to the M2 phenotype for the repair of damaged tissue and an anti-inflammatory response to return the tissue to its normal state prior to the injury. The M1 macrophage stage of a fresh wound usually takes between one and three days, with the M2 stage taking around four days [46]. Louiselle et al. [47] described the mechanism by which the M2 macrophage phenotype can enhance anti-inflammatory responses and accelerate the process of wound healing through the expression of the M2 phase, inducing factors such as RELM-α and Arginase 1 [47]. However, with diseases such as diabetes, the M1 phenotype is predominant regardless of the time after the initial injury. The inability to reach an M2 stage can lead to the greatly impaired ability of tissue repair and healing, resulting in an open wound that can lead to numerous complications, such as infection [47].

Macrophages develop an important role not only during the inflammation process but also because they are extremely heterogeneous and plastic cells, and their reversibility of polarization has a critical therapeutic value. Hence, we know that M1 (classically activated macrophages), which we have known as pro-inflammatory first responder cells, are polarized by LPS and can produce a large list of inflammatory cytokines such as IL-1β, IL-6, IL-12, IL-23 also TNF-α, either alone or associated with Th1 cytokines such as GM-CSF or IFN-γ [46]. Macrophage differentiation has proven to be a valuable therapeutic treatment for both diabetic and non-diabetic wounds.

## 3. Exosome Therapy

**Figure 3:**
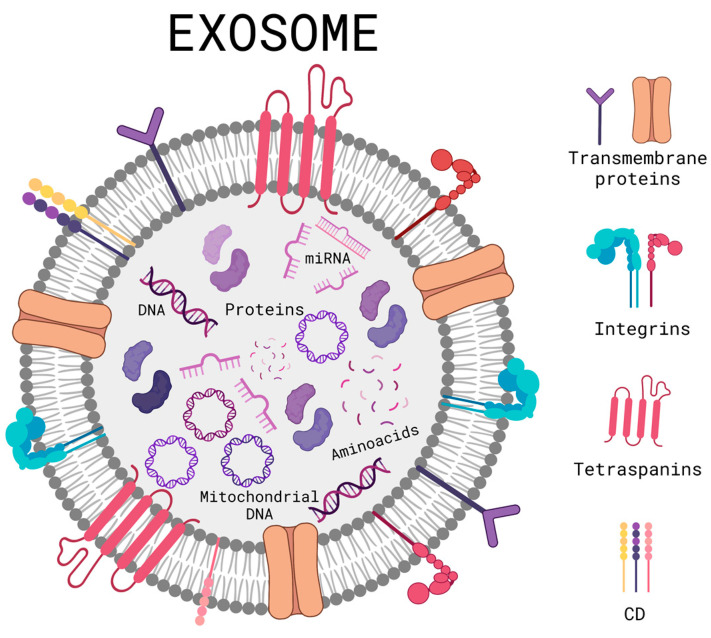
Schematic diagram of a general structure of an exosome. Created with BioRender.com (accessed on 2 June 2023).

As previously described, there are various skin disorders that can trigger immunosuppression, DNA mutations, ROS production, and inflammation; currently seeking alternatives that can combat these disorders and avoid their consequences. Extracellular vesicles are vesicles that are secreted by cells in order to communicate and, in turn, regulate physiological processes such as tissue repair. They have the advantage of being able to easily cross biological barriers such as the blood–brain barrier and the cell membrane. Within the extracellular vesicles, we found a subgroup called exosomes; they are nano-sized extracellular vesicles that typically range between 30 and 200 nm [48]. Exosomes are formed (Figure 3) by a lipidic bilayer that can accept both hydrophobic and hydrophilic drugs [49]. On their surface, they contain immune regulatory molecules, membrane proteins, and membrane trafficking molecules, which can help the exosome to attach or ignore the site of interest, turning them into selective extracellular vesicles for the delivery of the biomolecules contained inside, leading to cell communication. The exosome can transport mRNA, nucleic acids, or protein chaperones if they carry substances that are instead lipids, proteins or have a cytoplasmic content; they are accepted by recipient cells and can lead to the modification of pathological or physiological functions of the target cells as a result of exosome-specific cell interactions [50]. Exosomes deposit their content to target cells through different pathways, a ligand-receptor for subsequent activation of signaling pathways, pinocytosis, phagocytosis, and fusion with the plasma membrane [51].

The biogenesis of exosomes is based on the invagination of the surface of the cell membrane, and subsequently, primary endocytosis occurs; with the help of lipids, extracellular and membrane proteins are internalized to fuse and form early endosomes. Later they mature and become late endosomes, and, in this stage of maturation, they undergo more invaginations, achieving the specific encapsulation of certain nucleic acids and proteins, producing intraluminal vesicles and later becoming multivesicular bodies. Depending on the biomolecules encapsulated and expressed on the surface, they define the fate of these vesicles within or outside the cell; if their destination is inside the cell, they can be degraded by proceeding to the lysosomes and, on the other hand, if their destination is outside the cell, they first translocate to the membrane before being guided by RAB GTPase proteins with the dynamic support of cytoskeletal proteins. Then, they dock, and membrane fusion occurs, mediated by the N-ethylmaleimide-sensitive fusion attachment protein receptor (SNARE) family of soluble proteins, which subsequently expel them out of the cell and, when released, they are called exosomes [18,20,22]. However, multivesicular bodies do not always follow this route; they can deviate and generate hybrid molecules called “amphisomes” when fused with autophagosomes to later expel exosomes with a different biological load. This is one of the concerns and limitations of the use of exosomes as therapy, and the lack of heterogeneity can activate different undesired physiological processes [52].

Exosomes are commonly used for controlled drug delivery, biomarkers, tissue repairing agents, and even as an important part in the development of vaccines [49]. Exosomes have also been widely used as part of skin care products for anti-wrinkle, anti-aging, general hair, skincare, and wound healing. Exosomes can transport biologically active molecules such as nucleic acids, carbohydrates, proteins, antigens, and other molecules which can be used for different therapeutic treatments such as the regulation of skin pigmentation by using exosomes secreted by melanocytes or the influence of melanin production through exosomes secreted by skin cells through the mediation of paracrine, endocrine, and autocrine effects [53]. Exosomes can be harvested from several fluids and tissues of both human and animal origin, such as blood, urine, breast milk, saliva, urine, bile, and umbilical cord cells [48]. Depending on their parental cell, exosomes contain different biomolecules, such as proteins, lipids, or nucleic acids. The said cargo can also result from the exosome’s intercellular release site and the physiological condition of its cell [50].

### 3.1. Exosome Purification and Isolation

In order to use exosomes, they must be purified, removing unwanted molecules such as proteins, protein aggregates, and RNA granules, as these additional molecules can affect the exosome’s function, leading to unexpected effects. The technique used to separate these molecules can have an effect on the exosome’s functionality, either by damaging the exosomes themselves or by not properly eliminating certain compounds. These processes attempt to reduce the substance’s complexity due to its composition with a minimal loss of yield [54]. To isolate the exosomes, there are a plethora of methods, such as differential ultracentrifugation (DC), which are normally used to separate exosomes from biological fluids; this is based on the differences in size and density present within the exosomes and any unwanted molecules. This method has low effectiveness; the material obtained is highly heterogeneous, but its purity is notably low, and this process can also isolate unwanted molecules of a similar size and density as exosomes, affecting the compound’s function. The G-force applied alongside the centrifuge’s motor itself can also affect the result, as the process has low repeatability. Density-gradient centrifugation (DGC) is another purifying method; in addition to a centrifuge, a gradient of sucrose is used, which enables the separation of non-vesicular components. This process yields high purity while potentially isolating specific exosome subtypes. This method, however, can prove to be time-consuming, and there is a high risk of sample loss during the handling of the material. Density-cushioned ultracentrifugation (DCGC) can be used after DGC to ensure high purity in the resulting substance with large volume samples. Sedimentation velocity and buoyant density are considered to identify and eliminate unwanted molecules. Both the biological activity and physical integrity of the exosomes are maintained [55,56,57]. The affinity isolation method tags specific protein bodies on the substance, such as antibodies or peptides. Exosomes can be isolated from biofluids and cell culture media. This process yields the expected results; however, the surface proteins and the functionality of the exosomes might suffer damage. Other methods, such as polymer precipitation, have been used, and while this method is fast and suitable for large-volume samples [57], it yields a notably impure and easily contaminated solution, resulting in a less-than-ideal process [58].

### 3.2. Exosomes Derived from Mesenchymal Stem Cells (MSC-EXO) and Their Application in Different Skin Disorders

Mesenchymal stem cells (MSCs) can be isolated from different human tissues, such as umbilical cord blood or tissue, placenta tissue, and the spinal cord, and they are considered and called multipurpose cells [59]. This type of cells can differentiate into chondrocytes, osteocytes, and adipocytes, among others, depending on their origins, and because of this capacity and the way they can reproduce themselves, MSCs have gained the attention of researchers to start finding new treatments for different diseases [48]. MSCs are preferred for being a source of therapeutic exosomes due to their safeness and immunomodulatory properties [60]. MSCs secrete exosomes and other biomolecules that have been demonstrated to be beneficial in the repair of the attribute in paracrine signaling, which is the one that includes vesicles and exosomes [59]. These MSCs-EXOs contain different biomolecules that help them to perform specific functions such as lipids, proteins, and RNA, which play the role of maintaining their integrity and stability to enhance recognition and targeting on the surface and to improve their potential for controlling late translation and transcription receptors, respectively. MSCs-EXO present qualities similar to no other exosomes: they are small, have good biocompatibility, low immunogenicity, and have a long-circulating life. MSCs-EXO isolated from breast milk have shown stability for around 90 days [61], as opposed to other biomolecules used for drug delivery, such as neutrophils which have a much shorter life expectancy of around 16 h [62]. MSCs-EXO also penetrates easily, making them a great fit for drug delivery, immune therapies, and other therapies [48]. Due to these characteristics of exosomes, they can be used for various applications, including tissue regeneration and wound healing, as demonstrated by researchers using MSC-EXO obtained from placental umbilical cords [59]. In addition, MSC-EXO secondarily regulates the expression of growth factors, activating signaling pathways, such as phosphatidylinositol 3-kinase/protein kinase B (PI3K/AKT), the extracellular signal-regulated kinase cascade (ERK) or Wnt/β-catenin, resulting in re-epithelialization by cell proliferation and migration and angiogenesis [60].

MSCs-EXOs are considered of great interest due to their regenerative and immunomodulatory functions, leading to the development of anti-aging, wound healing, and anti-inflammatory treatments. The use of MSCs differs depending on the tissue from which they are originally extracted and can be used as regulators of immune responses through their differentiation into cells such as macrophages and natural killer cells [48]. Specific MSCs have proven their efficiency in treatments such as the use of ASC-exosomes when derived from adipose tissue in the treatment of atopic dermatitis [63] and Umbilical Cord Blood MSCs for the treatment of cutaneous wounds [64]. Therefore, it is important to consider the specific uses that different MSCs can have due to their tissue of origin.

The use of MSC-EXO has also been shown to lead to the polarization of the M2 macrophage phenotype, ensuring tissue repair, regardless of diseases such as diabetes. Ti et al. [65] found that human umbilical cord mesenchymal cells previously treated with lipopolysaccharides showed promising results through the regulation of the TLR4/NF-KB/STAT3/AKT signaling pathways and, specifically, TLR4 was downregulated while STAT3/AKT was upregulated. This model was applied to diabetic rats with cutaneous excisions, and after three days of exosome applications, the expression level of M2 macrophages ended up higher than M1 macrophages at the site of the wound [65]. Human bone marrow stromal cells have also shown great potential in the treatment of diabetic wounds through M2 macrophage polarization. Liu et al. [66] found that the inflammatory phase, or M1 macrophage phase, was inhibited by the expression of the PTEN gene, resulting in the activation of the PI3K and AKT pathways.

In the case of cutaneous injuries, several other mechanisms have been studied for their use of exosomes. Macrophage polarization has been used, as discussed previously; however, other methods were applied, such as the inhibition of white blood cells, keratinocytes, and fibroblasts, as well as the downregulation of the TLR4 pathway, which resulted in the lower production of inflammatory factors, enhancing the speed of wound healing [2,47,67].

Activating protein-1 (AP-1) refers to transcription factors, including activating transcription factor subunits: Jun and Fos dimers that bind to a specific site on DNA molecules known as the AP-1 binding site. Different factors regulate the transcription process of specific genes, leading to different physiological functions, such as keratinocyte proliferation and differentiation, cellular proliferation, apoptosis, and even cancerous transformation. The activity of AP-1 is up-regulated via the interaction of specific protein kinases such as MAPK and transcriptional coactivators. Protein interactions can also play an important role in a reduction in the transcription process. The activation of AP-1 activity can result in an extracellular stimulus or the exposure and activation of AP-1 proteins such as c-Jun, which, in turn, are regulated by the control of gene transcription. The function of activation proteins can also lead to the transcription of metalloproteinase genes, which degrade collagen and elastin, impairing the skin’s flexibility and elasticity [68].

MAPK can regulate AP-1, both during transcription processes and post-transcription, affecting the molecule’s ability to bind to DNA chains [69]. An increase in MAPK activity results in an increase in AP-1 activity, enhancing the ability of the transcription factors to bind to DNA molecules and trigger the transcription process, allowing the formation of RNA and, eventually, specific protein compounds [68]. On the other hand, molecules such as glycogen synthase kinase 3-β (GSK3-β) act as down regulators for AP-1 [70].

**Figure 4:**
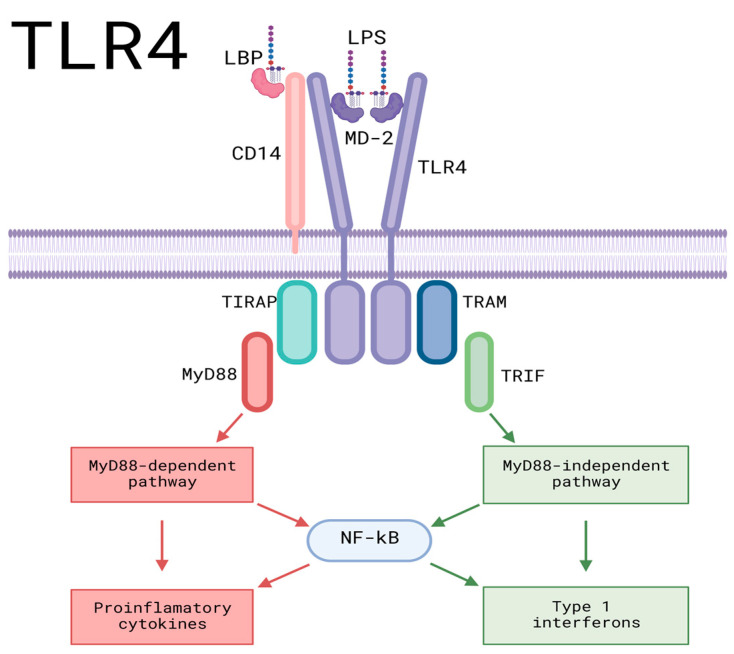
Pro-inflammatory signaling pathways of TLR4. LPS and LBP bind, leading the formed molecule toward CD-14, binding LPS and CD-14. It then interacts with the TLR4/MD-2 complex before being transported to the TLR4 monomers and leading to the dimerization of TLR4, activating intracellular TIR domains, which results in the activation of downstream proteins for signaling. TIRAP binds to the TIR domain, allowing the binding and activation of the myeloid differentiation molecule My-D88, leading to the My-D88-dependent pathway, which culminates in the activation of proinflammatory cytokines. TRAM can also bind to the TIR domain, resulting in the My-D88-independent pathway, culminating in the production of Type 1 interferons. AP-1: activating protein-1; CD14: cluster of differentiation 14; IKKs: IκB kinase complex; IL-1β: Interleukin-1 beta; IRAK-1: interleukin-1 receptor-associated kinase; IRAK-4: interleukin-4 receptor-associated kinase; IRF5: Interferon regulatory factor 5; IκBζ: NF-kappa-B inhibitor zeta; LBP: LPS-Binding Protein; LPS: Lipopolysaccharide; MAPK: Mitogen-activated protein kinase; MD-2: Myeloid Differentiation Factor 2; MyD88: Myeloid differentiation Factor 88; NFKB: Nuclear factor kappa B; TAK1: Transforming growth factor β-activated kinase 1; TIRAP: Toll-interleukin domain-containing adapter protein; TLR4: Toll-like receptor 4; TNF-α: Tumor Necrosis Factor-alpha; TRAF6: TNF receptor-associated factor 6; TRAM: Translocating chain-associating membrane protein; TRIF: TIR domain-containing adaptor-inducing interferon-β. Created with BioRender.com (accessed on 2 June 2023).

The MAPK signaling pathways represent pathways that activate and phosphorylate proteins, giving them the required energy to realize their function [71]. The pathway’s activation requires the binding of a ligand to a receptor known as RTK; the receptor catalyzes phosphorylation itself, then, growth factors bind to the phosphorylated RTK. Through a binding protein, a Ras molecule could be energized, exchanging its original GDP molecule with GTP and activating the protein. The activated Ras can then bind to effector proteins, such as B-Raf, which gives energy to MEK 1 and 2 via phosphorylation and, in turn, phosphorylate ERK 1 and 2. This can lead to the activation of the AP-1 factors Fos and Jun. Once activated, the factors bind to an AP-1 receptor in a chain of DNA, leading to the expression of different genes (Figure 4).

Gao et al. [25] reported that the levels of ERK, Jun, and Fos phosphorylation were elevated by UVB radiation, leading to increased transcription of MMPs and, specifically, MMP-1. To lower the production rate of MMPs, miR-1246-over expressing exosomes were used to suppress the activity of both the AP-1 and MAPK signaling pathways through the suppression of phosphorylated Fos and Jun production. The levels of p-ERK, p-Jun, and p-Fos were decreased by 66.5%, 70.6%, and 70.1%, respectively, greatly lowering the rate of MMP-1 transcription and the degradation of collagen and elastin through the manipulation of both the MAPK and AP-1 pathways. Li et al. [72] used a stem cell conditioned medium to facilitate organ and tissue repair through a decrease in the phosphorylation of Fos and Jun in the MAPK/AP-1 cascade, avoiding the transcription through AP-1 and the production of MMP1 along proinflammatory molecules such as IL-6.

On the other hand, considering the effects of UV radiation, MSCs-EXOs were used to counteract oxidative stress and skin aging by degrading MMPs and down-regulating the production of reactive oxygen species. Gao et al. [25] used miR-1246-overexpressing exosomes, which led to the reversal of the effects triggered by excessive UVB radiation, such as the production of reactive oxygen species, the secretion of MMPs, and the degradation of collagen. The transported miR-1246 led to the upregulation of TGF-β1, which promoted the synthesis of collagen and elastin. This molecule also leads to M2 macrophage polarization via the activation of the STAT3 signaling pathway.

Studies regarding in vitro models of keratinocytes damaged by oxidative stress with H_2_O_2_ and in vivo UV-irradiated mice, to which MSC-EXO was used, observed a decrease in ROS production and a decrease in radiation-induced DNA damage. In addition, aberrant calcium signaling and mitochondrial changes, as well as inflammation, were decreased. The authors demonstrated that MSC-EXO had antioxidant capacities, acting through the Nuclear factor erythroid 2-related factor 2 (NRF2) pathway; the size of the exosomes used could determine them as powerful nanotherapeutic agents against oxidative stress models [73].

Exosomes have been used in clinical trials for the treatment of various diseases, used as biomarkers, exosome vaccines, drug delivery systems, and exosome therapy [52]. The ClinicalTrials.gov website (https://clinicaltrials.gov/ accessed on 6 June 2023) lists different clinical trials, their current status, and the pathology for which they are used. A search was carried out with the term “exosome”, excluding conditions or diseases that were not related to the skin, and the following results were shown hair loss, alopecia, psoriasis, squamous cell carcinoma of the head and neck, melanoma, diabetic foot, wounds, and injuries. One of the clinical trials with already published results was the use of exosomes to treat patients with stage III/IV metastatic melanoma, with the inclusion criteria of HLA-DPO4+, -B35+, or HLA-A1+, stage IIIB, and IV leukocyte phenotype, and tumors expressing the MAGE3 antigen. Autologous exosomes, isolated from dendritic cells, were used to immunize the patients. Their results showed no adverse effects, in addition to tumor regression in lymph nodes and skin lesions, as well as the depigmentation of the naevi and loss of the tumor’s class I major histocompatibility complex [74].

To use exosomes in clinical trials, good manufacturing practices must be followed, which are governed by safety concerns; these practices monitor and divide the process into three major steps: the production, purification/isolation, and characterization of the exosome, always maintaining sterility and ensuring the quality and heterogeneity of the exosomes. For the production process, different types of cells can be used, such as MSCs, dendritic cells derived from monocytes, and adipose tissue-derived stem cells (ADSC), among others, in order to induce the formation and release of exosomes, the system, the environment, and the cell culture medium, as well as the dissociation enzyme, which must be monitored. As mentioned in Section 3.1, there are several methods to purify and isolate exosomes. In general, terms, what regulates the good manufacturing practices of this process is the elimination of unwanted cell debris, the concentration of the conditioning medium, and the isolation of exosomes. The last step is the characterization of the exosome, which is based on ensuring that exosomes are isolated through validation or biological and physicochemical characterization, measuring bioactivity, observing structure, and finally, measuring adsorption and protein content [75].

Therapies based on MSC-EXO have been shown to be promising therapy for skin repair and regeneration, depending on the application and the source. In Table 1, we summarize a variety of uses for MSC-EXO obtained from various sources with different models that demonstrate the effects of treating skin damaged by UV, diabetic wounds, and cutaneous and burn-related injuries.

## 4. Signaling Pathways Involved in Exosome-Mediated Skin Regeneration

### 4.1. MSC-EXO and TLR Signaling Pathway

Toll-like receptor proteins are fundamental components of the immune system; they are stimulated by the presence of bacteria, viruses, and fungi that have the potential to harm the organism. Each TLR is stimulated by specific pathogens that can enter the skin through a wound. TLR interactions can result in inflammatory responses through the expression of proinflammatory cytokines, and, in the case of the MY-D88-dependent pathway, TLR can result in a different pathway, the TRIF-dependent pathway, which leads to the production and activation of type-1 interferons [92].

TLR4 is stimulated by molecules that are rich in LPS, such as Gram-negative bacteria and even tumoral viruses. LPS is an important component of bacteria, as it can lead to the induction of systemic inflammation and even sepsis [67]. The pathway is formed by two TLR4 monomers within the cell membrane, which are both associated with myeloid differentiation factor 2 (MD-2). The cell membrane contains two clusters of differentiation 14 (CD-14). The pathway itself begins when LPS binds to LBP and the LPS binding protein, which leads the molecule towards CD-14, binding LPS and CD-14. This substance then interacts with the TLR4/MD-2 complex. LPS is then accepted by MD-2 and transported to the TLR4 monomers, which leads to the dimerization of TLR4, activating intracellular TIR domains, which, in turn, activates the downstream proteins for signaling. TIRAP, an adaptor protein, then binds to the TIR domain, allowing the binding and activation of the myeloid differentiation molecule My-D88, leading to one of the possible TLR-4 pathways [93] and resulting in a proinflammatory response through the release of inflammatory factors such as IL-1β and TNF-α (Figure 4) [85].

This pathway leads to the activation of IL-1 receptor-associated kinase-4 (IRAK-4): a molecule of great importance for the transmission of TLR signals and the production of proinflammatory cytokines. IRAK-1 is then activated and bonded with an adaptor protein, TRAF6, which forms a complex with two enzymes: UBC13 and UEV1A, activating transforming growth factor-b-activated kinase 1 (TAK1). TAK1 then activates the IKB kinase (IKK) and MAPK pathways. The IKK pathway results in the translocation of the transcription factor NF-KB, which is responsible for the control of proinflammatory cytokine expression (Figure 4) [93].

It has been shown that exosome-transported let-7b microRNA levels of TLR4 were reduced to reduce the inflammatory response when using exosome-transported let-7b microRNA [65]. The expression of the pathway and NF-KB resulted in decreased inflammation in an in vivo mouse model through the targeting of 15 and 14 genes within the TLR and NF-KB pathways, respectively [94].

In a burn-induced mouse model, the use of human umbilical cord mesenchymal stem cell exosomes resulted in the suppression of the TLR4 pathway, preventing the release of inflammatory factors such as TNF-α and IL-1β by overexpressing miR-181c, and significantly reducing post-injury inflammation. The miR-181c molecule could inhibit the TLR4 signaling pathway by binding to the 3’ untranslated region of TLR4 and restricting the inflammatory response of the cascade [82].

### 4.2. MSC-EXO and NRF2-KEAP1 Pathway

**Figure 5:**
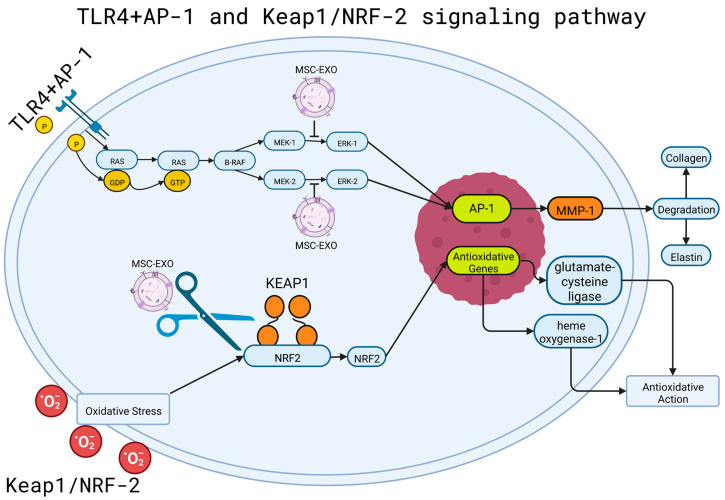
TLR4 + AP-1 and keap1/NRF-2 mechanisms of action and stem cell disruption. Shown in the top portion is the MAPK pathway, which activates and phosphorylates proteins. A Ras molecule is energized, exchanging its original GDP molecule with GTP, activating the protein. The activated Ras binds to B-Raf, an effector protein, which gives energy to MEK 1 and 2 via phosphorylation, and, in turn, phosphorylates ERK 1 and 2. This can lead to the activation of the AP-1 factors Fos and Jun. Once activated, the factors bind to an AP-1 receptor in a chain of DNA, leading to the expression of different genes and the production of MMP-1, which causes the degradation of elastin and collagen. MSC-EXO can be used to interrupt the activation of ERK 1 and 2, inhibiting the activation of AP-1 and, in turn, the degradation of elastin and collagen. The bottom portion shows the NRF2-KEAP1 pathway, triggered by oxidative stress. Traditionally, NRF2 and KEAP1 regulate each other, leading to the production of anti-inflammatory factors in response to oxidation. MSC-EXO can sever the connection between the two molecules, disrupting the production of anti-inflammatory factors. AP-1: Activating Protein-1; B-RAF: B subunit proto-oncogene serine/threonine-protein kinase; ERK-1/2: Extracellular signal-regulated kinase 1/2; GDP: Guanosine diphosphate; GTP: Guanosine Triphosphate; KEAP-1: Kelch-like ECH-associated protein 1; MEK-1/2: Mitogen-activated protein kinase kinase 1/2; MMP-1: Matrix metalloproteinase-1; MSC-EXO: mesenchymal stem cell exosome; NRF-2: Nuclear factor erythroid 2-related factor 2; P: Phosphate; RAS: rat sarcoma family of small G proteins. Created with BioRender.com (accessed on 2 June 2023).

The NRF2-KEAP1 pathway is the main defensive response triggered by oxidative stress, which regulates the transcription of antioxidant genes to eliminate the possible damage caused by oxidation and any existent carcinogens [94]. KEAP1 regulates the activity of NRF2 through the ubiquitination and degradation of the transcription factor, which is responsible for the regulation of responses to oxidative stress. NRF2 is then left in an inactivated state without external stimuli [95]. As a response to stress, KEAP1 is inactivated, leading to NRF2 and avoiding ubiquitination, accumulating in high quantities within the cell and translocating to the nucleus, leading to the promotion of secondary antioxidative action [96]. The overexpression of KEAP1 results in the repression of NRF2’s transcriptional activity; by contrast, the absence of KEAP1 leads to the activation of NRF2 and its response to oxidative stress. A change in the interaction between KEAP1 and NRF2 could lead to the activation of the NRF2 pathway, resulting in the activation of anti-inflammatory factors such as glutamate-cysteine ligase and heme oxygenase-1 [95]. One of these cases occurred due to the action of the kinase inhibitor p21Cip/WAF1, which is bound to NRF2 in the place where KEAP1 usually binds, disrupting the regular interaction between the two molecules. Another disruptor to the interaction is p62, which was upregulated in the presence of oxidative stress, resulting in the activation of NRF2 and the antioxidant defense triggered by the molecule (Figure 5) [94].

Once separated from KEAP1 and translocated to the nucleus, NRF2 heterodimerizes with Maf proteins, which grant the molecule the ability to upregulate the electrophile response element (EpRE)-mediated transcription, results in the transcription of genes that contain an antioxidant response element (ARE) in their promoter section [97]. Wang et al. [87] used stem cell-derived exosomes to regulate the activity of the NRF2 defense system in a mice model with UV-induced oxidative stress. This study showed that oxidative stress increased NRF2 signaling, regulating stress and inflammatory response. The MSC-EXO used resulted in the regulation of the defense system and the diminishing of oxidative stress-induced skin injuries (Figure 5) [87].

### 4.3. MSC-EXO and PI3K/Akt Pathway

Skin homeostasis was regulated by the PI3K/Akt pathway. Therefore, dysregulation of this signaling pathway ked to the development of malignant skin disorders such as melanoma, both basal cell carcinoma (BCC) and squamous cell carcinoma (SCC), skin tumors, and non-malignant disorders such as psoriasis, acne, vitiligo, scleroderma, and alopecia, among others [98].

The serine/threonine kinase Akt was considered a proto-oncogene, which could be activated by different factors such as integrins, receptor tyrosine kinases, T and B cells, coupled to G proteins, and phosphatidylinositol (PI3K) [98]. Within this signaling pathway, there was a regulatory PTEN molecule, which could be involved in improving cell sensitivity to apoptosis and inhibiting cell proliferation. To activate PTEN, it was necessary to use PIP3, the latter of which could be dephosphorylated to pass to PIP2 and decrease the action of this pathway; therefore, if PTEN was inactivated, the sustained activation of PI3K/Akt could be achieved. In turn, if AKT was activated, it could activate mTORC1 resulting in cell proliferation and growth. Another effect was to phosphorylate TSC2 by inhibiting it for its subsequent activation of mTORC1 indirectly through Rheb-GTP. Undesirably, the activation of this pathway could increase the expression of MMP-2, whose function is the degradation of the extracellular matrix and promotes metastasis.

**Figure 6:**
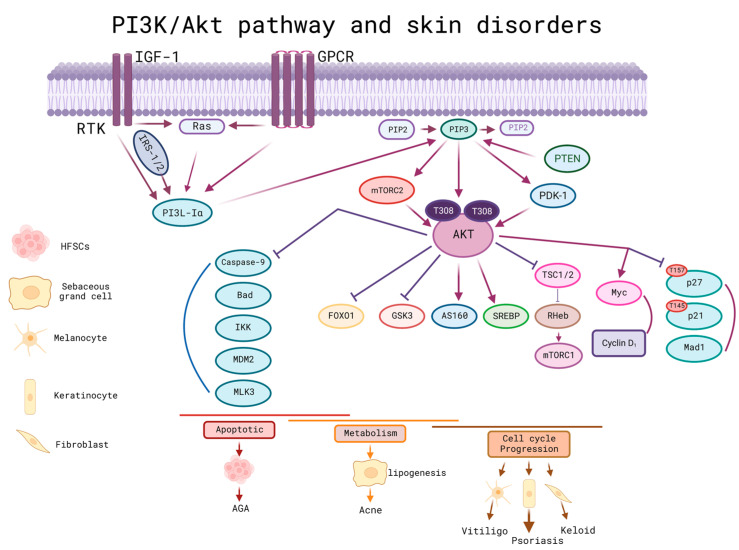
PI3K/Akt pathway and its relationship with skin disorders/diseases. IGF-1 and GPCR are the first step, followed by the activation of the classical PIP3 pathway to activate AKT; the PTEN regulatory molecule is activated by PIP3 and can be dephosphorylated to pass to PIP2 and decrease the action of this pathway; therefore, if PTEN is inactivated a sustained activation of PI3K/Akt could be achieved. In turn, if AKT was activated, it could activate mTORC1 resulting in cell growth and proliferation. Another effect was to phosphorylate TSC2 by inhibiting it for its subsequent activation of mTORC1 indirectly through Rheb-GTP. Indirectly, the activation of this pathway could increase the expression of MMP-2, whose function is the degradation of the extracellular matrix and favors metastasis. FOXO is a protein downstream of this pathway; it is a mediator of non-malignant skin disorders, responsible for the control of the cell cycle, apoptosis, longevity, and healing, and involved in the inhibition of lipogenesis. This protein can give rise to disorders such as acne, psoriasis and promote the apoptosis of keratinocytes. AGA: Androgenic alopecia; AKT: protein kinase B; FoxO1: Forkhead box O1; GPCR: G-protein-coupled receptors; GSK3: glycogen synthase kinase 3; IGF-1: Insulin-like growth factor 1; IKK: IkB-kinase; IRS-1/2: insulin receptor substrate-1/2; MAD1: MAX dimerization protein 1; MDM2: murine double minute 2; MLK3: mixed lineage kinase 3; mTORC1/2: mTOR complex 1/2; PDK1: phosphoinositide-dependent protein kinase 1; PI3K: phosphatidylinositol 3-kinase; PIP2: phosphatidylinositol 4,5-biphosphate; PIP3: phosphatidylinositol 3,4,5-triphosphate; PTEN: Phosphatase and tensin homologue; RTK: receptor tyrosine kinases; SREBP: sterol regulatory element-binding proteins; TSC1/2: tuberous sclerosis complex 1/2. Created with BioRender.com (accessed on 2 June 2023).

In non-malignant skin disorders, one of the proteins involved in pathophysiology is called FOXO; this protein is a mediator of the gene transcripts responsible for cell cycle control, apoptosis, longevity, and healing, among others. It is a downstream factor for the PI3K/Akt signaling pathway (Figure 6) [91]. In acne, this pathway is involved in the inhibition of lipogenesis through the activation and nuclear export of the FOXO1 protein [89,90,91]. In addition, Akt overactivation could be induced by PTEN depletion, which can contribute to psoriasis by promoting abnormal proliferation and the apoptosis of keratinocytes [99].

Within other non-malignant skin disorders, exosomes in platelet-rich plasma (PRP) have been shown to be a promising treatment for alopecia as they promote the survival of hair follicle stem cells by activating the cascade pathway. Akt/Bad. In this sense, exosome therapy can activate the PI3K/Akt pathway, regenerating and maintaining the hair follicle [100].

Thanks to ADSC, significant results have been achieved in accelerating wound healing. ADSCs were shown to promote cell proliferation and migration in vitro when taken up by fibroblasts thanks to the increased levels of type I and III collagen, bFGF, and TGF-β1. In vivo mouse models activated the p-Akt/Akt signaling pathway. In summary, ADSCs can accelerate wound healing by the deposition of collagen in the PI3K/Akt signaling pathway [101].

## 5. The Role of Exosome in Epigenetics Processes Related to Cell Proliferation, Cell Migration, Differentiation, and Modulation of the Inflammatory Response

Epigenetics refers to heritable changes in gene expression that do not involve alterations in the DNA sequence. These changes can be caused by various factors, such as chemical DNA modifications or the histones that surround it [102]. The epigenetic code encompasses covalent modifications to the DNA or its associated proteins, including the addition of functional groups (methyls, acetyls, phosphates) or proteins (ubiquitin, SUMO). These modifications have implications for the transcriptional regulation of genes, with some activating or inhibiting the transcription and impacting how genes are read and expressed [103]. Numerous studies have highlighted the significance of chromatin remodeling and histone modifications in the sequential recruitment of proteins to DNA, allowing the strict control of gene expression [104]. Chromatin remodeling can be induced by post-translational modifications of histones, such as phosphorylation, acetylation, methylation, ubiquitination, and sumoylation, at various Lys, Ser, or Arg residues located in the amino-terminal region of histones, which directly interact with DNA. Enzymes associated with transcription activating or repressing complexes affect these histone modifications [105]. Other molecules involved in epigenetic processes include microRNAs (miRNAs), which are small non-coding RNAs, approximately 18 to 25 nucleotides in length, that participate in the epigenetic regulation of transcription. miRNAs are epigenetic modulators that affect the protein levels of target mRNAs without modifying gene sequences. Furthermore, miRNAs themselves can be regulated by epigenetic modifications, including DNA methylation, RNA modification, and histone modifications. miRNAs play critical roles in various biological processes such as cell proliferation, differentiation, apoptosis, and haematopoiesis [106].

Exosomes play an important role in modulating epigenetic processes; they have the ability to transfer RNA and proteins to recipient cells, thereby influencing gene expression and cellular processes. The ability of stem cells to repopulate the epidermis may depend on the underlying epigenetic landscape. As our understanding of these changes improves, new epigenetic-based approaches may emerge to enhance the regenerative potential of epidermal stem cells. Multiple epigenetic mechanisms are involved in epidermal stem cell differentiation, including DNA methylation, histone acetylation, the regulation of transcriptional coactivators and corepressors, as well as non-coding RNAs [106]. In the case of human primary keratinocyte differentiation, DNA methylation can play a significant role. Methylation in the promoter site of the TRIM29 gene, for instance, can lead to the lower expression of genes involved in the terminal differentiation of keratinocytes (Smits et al., 2020). Additionally, long non-coding RNA COL1A2-AS1 was found to limit fibroblast growth during wound healing by promoting apoptosis through the repression of p-SMAD3 gene expression and the activation of beta-catenin gene expression. This is relevant when controlling excessive fibroblast proliferation in keloid scarring [107]. In the case of human dental follicle stem cells (DFSC), it has been observed that EZH2 (Enhancer Of Zeste 2 Polycomb Repressive Complex 2 Subunit) promotes the Wnt/β-catenin signaling pathway by modulating the level of trimethylation in the lysine residue 27 of histone H3 (H3K27) and in the genes associated with these pathways [108].

Furthermore, several reports indicate that histone methylation plays a role in macrophage polarization. For instance, H3K27 methylation regulates the expression of the JMJD3 protein, which has roles in the activation of proinflammatory and anti-inflammatory macrophage phenotypes [109]. Histone H3K4 can be methylated by various members of the protein family that possess the SET domain. Among them, the MLL1 protein plays a crucial role in the repair of normal tissues. During the inflammatory phase of wound healing, MLL1 catalyzes the deposition of the H3K4me3 methylation mark on proinflammatory genes in macrophages, thus contributing to the tissue repair process [110].

Molecules such as miRNAs have the ability to promote chromatin remodeling, which can have an impact on the activity of various genes that are associated with skin regeneration. In a recent study, miRNA-34a was found to have a modulatory effect on the proliferation and differentiation of human MSC. Additionally, miRNA-34a was found capable of reversing the effects of proinflammatory cytokines and stimulating osteogenic differentiation, thereby promoting the regeneration of bone tissue [108].

Furthermore, studies have shown that mesenchymal stem cell-derived exosomes loaded with specific microRNAs have a synergistic effect on diabetic wound healing in vitro models. In a study conducted on mice, Gondaliya et al. [111] demonstrated that exosomes derived from mesenchymal stem cells, and loaded with an inhibitor of miRNA-155, exhibited synergistic effects on keratinocyte migration, including the restoration of FGF-7 levels and anti-inflammatory action. This resulted in accelerated wound healing through the negative regulation of miRNA-155 [111].

Another recent study focused on glomerular mesangial cells from diabetic rats treated with exosomes derived from ADSC loaded with miR-125a. The objective was to restore the function of genes involved in cell repair and regeneration mechanisms. The findings indicated that exosomes derived from ADSC had the ability to inhibit the expression of histone deacetylase 1 (HDAC1) and endothelin-1 (ET1). HDAC1 was a direct target of miR-125a, while ET1 was an intrinsic vasoconstrictor of the vascular endothelium. These results suggest the beneficial effect on both cell regeneration and the reduction in inflammation [112].

Exomes can promote skin regeneration by influencing cell proliferation, cell migration, differentiation, and the modulation of inflammatory responses through modifications in the epigenetic state of stem cells, epidermal cells and the modulation of the expression of genes that are involved in these mechanisms. Although research in this field is still limited and further study is needed, stem cell-derived exomes and epigenetics represent a promising area for the development of skin regenerative therapies and a personalized approach to regenerative medicine in the future.

## 6. Conclusions

Exosomes have therapeutic properties and applications in the regeneration and repair of skin tissue; they contribute to the regulation and promotion of the healing process by activating different pathways that can modulate the state of inflammation, promoting angiogenesis, proliferation, differentiation, cell migration, and apoptosis, and ensuring that the regular healing process takes place in situations it normally would not, such as diabetes, which affects the body’s natural healing and scarring processes, inhibiting its ability to recover the shape and function of the affected tissue. The pathways involved in exosome-mediated skin regeneration include MAPK, NRF2-KEAP1, AKT/mTOR, PI3k/AKT, ERK 1/2, and Wnt/β-catenin, among others. The use of stem cell-derived exosomes represents a promising strategy for the repair, regeneration, and treatment of malignant and non-malignant skin disorders.

## Figures and Tables

**Table 1:** Therapies with MSC-EXO for the repair and regeneration of the skin.

Exosome Source	Model	Mechanism of Action	Type of Application	Reference
Human umbilical cord	In vitro (THP-1) + LPS + rats + STZ	MSCs switched macrophages to an M2 state. Macrophage polarization was regulated through TLR4/NF-KB/STAT3/AKT regulatory signaling pathways.	Diabetic wound healing	[65]
Menstrual blood sample.	C57BL/6 + STZ	The M2 stage was induced through the downregulation of TLR4 and NF-KB. STAT3 and STAT6 were activated, triggering the expression of M2 genes such as Arg-1 and Arg-2.	Promoting wound-healing processes in diabetic wounds.	[76]
hBMSCs	SD rats + STZ	The activation of the AKT/PI3K pathways was inhibited due to the promotion of the expression of the PTEN gene. This lead to the inhibition of the inflammatory phase, leading to a quicker tissue regeneration phase. M1 polarization of macrophages was inhibited, leading to M2.	Diabetic wounds	[66]
Synovial membrane	HMEC-1 cells cultured in MCDB131 + 10% FBs + 10 ng/mL epidermal growth factor + 2 mM L-glutamine + 1 mg/mL hydrocortisone. Adult male Sprague Dawley rats.	An increase in the granulation tissue and angiogenesis. Using SMSCs and overexpressing miR-126-3p increased the strength of exosomes (SMSC-126-Exos); it was found that they could trigger the generation of newly formed vessels and also increase their maturation.	Wound healing in diabetic skin	[77]
ADSCs	BALB/c mice + high fat diet + STZ + skin injury	Fibroblast proliferation and migration were promoted through the activation of the PI3K/AKT pathway, accelerating the healing of diabetic wounds. TGF-β levels were also enhanced, stimulating fibroblast proliferation.	Promotion of diabetic wound healing	[78]
PGZ-exos + HUVECs + BMSCs	Mice + STZ + cutaneous wounds	The expression of p-AKT and p-PI3K was promoted, activating the PI3K/AKT/eNOS pathway and enhancing angiogenesis. PGZ-exos promoted collagen deposition, and re-epithelization accelerated wound healing.	Promotion of diabetic wound healing	[79]
hUCMSCs	SD rats + STZ + skin wounds	β-catenin activation in endothelial cells was induced, resulting in the promotion of wound healing. PF-127 hydrogel served as a vessel for the continuous release of exosomes.	Promotion of diabetic wound healing	[80]
Bone marrow from human jaw and iliac crest	C57BL/6J female adult mice + skin excision	Exosomes enhanced anti-inflammatory responses by secreting miR-223, which led to the M2 polarization of macrophages, accelerating wound healing. The expression of M2 factors RELM-α and Arginase 1 increased.	Dermal wound healing.	[81]
hucMSCs	Balb/C mice + excisional wound + peroxide	Stem cells inhibit cell-induced death by suppressing the translation of AIF and the hyperactivation of PARP-1, inhibiting the action of keratinocytes and fibroblasts, which accelerate the process of wound regeneration. CK14’s expression was upregulated, leading to improved integrity in the recently formed epidermis.	Cutaneous wounds	[73]
Human umbilical cord: hSFCs and hUCMSCs	SD rats + full thickness burn wounds	hUCMSC exosomes reduced the presence of white blood cells and the suppression of the TLR4 pathway, preventing the release of inflammatory factors such as TNF-α and IL-1β.	Reducing inflammation resulting from severe burns.	[82]
FDMSC sand ADFs	BALB/c mice + PBS + FDMSC-exosomes	Activating Notch Signalling using MSC-derived exosomes.	Cutaneous wound healing	[83]
Bone marrow mesenchymal stem cells of C57BL/6J mice and human umbilical vein endothelial cells	C57BL/6J + pentobarbitone sodium + dorsal wounds	The expression of p-AKT and p-eNOS was enhanced, stimulating angiogenesis through the activation of the AKT-mediated VEGF pathway.	Cutaneous wounds	[64]
hUCMSCs	SD rats + skin deep second-degree burn wounds	The number of epidermal and dermal cells significantly increased, and the rate of re-epithelialization was determined by the enhanced expression of CK19: an epithelial biomarker. PCNA-positive cells were found in the wound area, indicating the proliferation of cells. Exosomes deliver Wnt4, which activates Wnt/b-catenin and inhibits stress-induced skin cell apoptosis by the activation of the AKT pathway.	Accelerating re-epithelialization in burn wounds	[84]
Human umbilical cord blood stem cells	SD rats + excisional wound	TGF-β receptors are inhibited via miR-21-5p, and miR-125b-5p, resulting in a lower myofibroblast differentiation, preventing excessive scar formation. Collagen formation is inhibited in the late stages of the healing process.	Scar tissue	[85]
Primary human adipose stem cells	In vitro (HDFs) + UVB radiation	MMP production is downregulated, proliferating elastin, TGF-β1, TIMP-1, and collagen types I, II, III, IV, and V, assisting in the recovery of damaged fibroblasts.	Promoting the healing of dermal tissue damaged by UV radiation	[86]
hUCMSCs	Mice + UV radiation	The NRF2 (antioxidant defense system) could be regulated, resulting in an enhanced rate of repair of oxidative stress-induced skin injury. Collagen fiber density and epidermal thickness were increased. MSCs restored the calcium concentration in damaged keratinocytes. The expression of NRF2 signaling was decreased, resulting in the mediation of oxidation and inflammation factors.	Treatment of oxidative stress injuries resulting from UV radiation	[87]
Bone marrow derived mesenchymal stem cells	Dermal fibroblasts/mice + UVB radiation	UV-induced MMP1 and MMP9 expressions were greatly diminished. Type I procollagen synthesis was enhanced. MSCs produced growth factors such as VEGF, EGF, IGFBP, and bFGF, which contributed to angiogenesis and injury repair.	Diminishing the effects of aging and UV radiation such as poor collagen production	[88]
Human umbilical cord mesenchymal stem cells	Mice + acute skin photodamage	The expression of p-p65 was significantly decreased, and the levels of PCNA expression were increased. The γH2AX and 8-OHDG markers for DNA damage expression were lowered. The proliferation of HaCaT cells was enhanced, and apoptosis was inhibited. SIRT1 expression was also enhanced by the delivery of the 14-3-3ζ protein, leading to the promotion of cell survival via autophagy.	Prevention and repair of oxidative and UV-related damage	[89]
Human umbilical cord mesenchymal cells	Fibroblasts + UVB radiation	SA-β-gal-positive cells were reduced, indicating a lower rate of cell aging. The expression levels of MMP1 were decreased, while the expression of Col-1 increased, which suggested the recovery of regular cellular functions. The expression of the GPX-1 gene was upregulated, possibly leading to an increase in antioxidant activity.	Development of anti-aging treatment on dermal fibroblasts affected by UVB-induced photoaging	[90]
Adipose-derived stem cells	Human skin fibroblasts/mice + UVB radiation	The expression of MMP-1 was significantly reduced by the inhibition of the MAPK/AP-1 pathway. Exosomes rich with miR-1246 increased the gene expression of TGF-β1, which led to the promotion of collagen and elastin synthesis, as well as the transcription of type I procollagen.	Reducing the effects of photoaging derived from UVB radiation damage.	[25]
Colostrum of cows and commercialized milk	HDF + UV, HaCaTs + UV	Col M-exo transport	Damaged skin by UV light	[91]

ADF: Adult Dermal Fibroblasts; ADSCs: Adipose stem cells; AKT: protein kinase b; AIF: apoptosis-inducing factor; bFGF: basic fibroblast growth factor; BMSCs: bone marrow derived mesenchymal cells; CS: Chitosan; ERK1/2: extracellular signal-regulated kinase 1/2; eNOS: endothelial nitric oxide; EGF: epidermal growth factor; FBs: Fibroblasts; FDMSCs: Fetal Dermal Mesenchymal Cells; HaCaT: human epidermal keratinocyte; HASCs: human adipose stem cells; hBMSCs: human bone marrow stromal cells; HDF: Human dermal fibroblast; hESCs: human embryonic stem cells; HMEC-1 cells: Human dermal microvascular endothelial cells; hSFCs: human skin fibroblast cells; hUCMSCs: human umbilical cord mesenchymal stem cells; HUVECs: human umbilical cord vein vascular endothelial cells; LPS: lipopolysaccharides; IGFBP: insulin-like growth factor binding protein; IL-10: Interleukin-10; MAPK: mitogen-activated protein kinases; MenSCs: Menstrual blood-derived stem cells; MMPs: metalloproteinases; MSC: mesenchymal stem cells; NRF-2: Nuclear factor erythroid 2-related factor 2; PARP-1: Poly(ADP-Ribose) Polymerase 1; PBS: Phosphate Buffer Saline; PCNA: Proliferative cell nuclear antigen; PGZ-exos: exosomes pretreated with pioglitazone; SHED: Stem cells from human exfoliated deciduous teeth; STAT3: Signal transducer and activator of transcription 3; STZ: streptozotocin; VEGF: vascular endothelial growth factor.

## Data Availability

Not applicable.
